# DNA methylation of *GFI1* as a mediator of the association between prenatal smoking exposure and ADHD symptoms at 6 years: the Hokkaido Study on Environment and Children’s Health

**DOI:** 10.1186/s13148-021-01063-z

**Published:** 2021-04-07

**Authors:** Kunio Miyake, Chihiro Miyashita, Atsuko Ikeda-Araki, Ryu Miura, Sachiko Itoh, Keiko Yamazaki, Sumitaka Kobayashi, Hideyuki Masuda, Tadao Ooka, Zentaro Yamagata, Reiko Kishi

**Affiliations:** 1 Departments of Health Sciences, Interdisciplinary Graduate School of Medicine and Engineering, University of Yamanashi, 1110 Shimokato, Chuo, Yamanashi 409-3898 Japan; 2 Center for Environmental and Health Sciences, Hokkaido University, Hokkaido, Japan

**Keywords:** ADHD, Birth cohort, DNA methylation, DOHaD, *GFI1*, Prenatal smoking exposure

## Abstract

**Background:**

Prenatal smoking exposure has been associated with childhood attention-deficit/hyperactivity disorder (ADHD). However, the mechanism underlying this relationship remains unclear. We assessed whether DNA methylation differences may mediate the association between prenatal smoking exposure and ADHD symptoms at the age of 6 years.

**Results:**

We selected 1150 mother–infant pairs from the Hokkaido Study on the Environment and Children’s Health. Mothers were categorized into three groups according to plasma cotinine levels at the third trimester: non-smokers (≤ 0.21 ng/mL), passive smokers (0.21–11.48 ng/mL), and active smokers (≥ 11.49 ng/mL). The children’s ADHD symptoms were determined by the ADHD-Rating Scale at the age of 6 years. Maternal active smoking during pregnancy was significantly associated with an increased risk of ADHD symptoms (odds ratio, 1.89; 95% confidence interval, 1.14–3.15) compared to non-smoking after adjusting for covariates. DNA methylation of the growth factor-independent 1 transcriptional repressor (*GFI1*) region, as determined by bisulfite next-generation sequencing of cord blood samples, mediated 48.4% of the total effect of the association between maternal active smoking during pregnancy and ADHD symptoms. DNA methylation patterns of other genes (aryl-hydrocarbon receptor repressor [*AHRR*], cytochrome P450 family 1 subfamily A member 1 [*CYP1A1*], estrogen receptor 1 [*ESR1*], and myosin IG [*MYO1G*]) regions did not exert a statistically significant mediation effect.

**Conclusions:**

Our findings demonstrated that DNA methylation of *GFI1* mediated the association between maternal active smoking during pregnancy and ADHD symptoms at the age of 6 years.

**Supplementary Information:**

The online version contains supplementary material available at 10.1186/s13148-021-01063-z.

## Background

The concept of Developmental Origins of Health and Disease (DOHaD) suggests that exposure to environmental stressors during prenatal and early postnatal periods increases susceptibility to adverse health outcomes later in life. It is particularly well known that prenatal smoking exposure can cause adverse health effects not only at birth, but also in the long term after birth. For instance, prenatal smoking exposure increases the risk of several adverse birth outcomes, including infant death [1], preterm birth [2], and low birth weight [3, 4]. Furthermore, prenatal smoking exposure has been associated with child health implications, including obesity [5], asthma [6], and neurodevelopmental disorders such as antisocial behavior, conduct disorder, pervasive developmental disorder (PDD), and attention-deficit/hyperactivity disorder (ADHD) [7, 8].

DNA methylation, one of the epigenetic modifications, plays an important role in the regulation of gene expression. DNA methylation patterns are erased after fertilization and re-established during embryonic development [9]. DNA methylation is susceptible to several environmental factors, particularly during fetal life [10, 11]. In recent studies, prenatal smoking exposure has been associated with DNA methylation changes in cord blood. Previous epigenome-wide association studies (EWAS) have identified CpG sites of several genes, including aryl-hydrocarbon receptor repressor (*AHRR*), cytochrome P450 family 1 subfamily A member 1 (*CYP1A1*), growth factor-independent 1 transcriptional repressor (*GFI1*), and myosin IG (*MYO1G*), that are sensitive to maternal smoking exposure [12–14]. Several studies have reported that DNA methylation changes are involved in prenatal smoking exposure and low birth weight. DNA methylation differences of several genes, including *AHRR*, *GFI1*, and *EXOC2*, are significant mediators between prenatal smoking and low birth weight [15–17]. In contrast, there remain few reports regarding the effects of DNA methylation on the association between prenatal smoking exposure and postnatal outcomes.

ADHD is a complex disease that interacts with genetic and environmental factors. In addition to genetic approaches, recent studies have identified the epigenetic alterations involved in ADHD. Several CpG sites that are differentially methylated between cases and controls have been identified by candidate-gene and EWAS approaches [18–20]. In addition, two birth cohort studies have reported an association between DNA methylation at birth and childhood ADHD. Walton et al. [21] demonstrated that DNA methylation at birth relates to later ADHD symptom trajectories, although such an association was not observed at the age of 7 years. Gervin et al. [22] demonstrated that long-term prenatal exposure to paracetamol (acetaminophen) is associated with DNA methylation differences in children diagnosed with ADHD. Prenatal factors such as maternal substance (e.g., tobacco, alcohol, and maternal anti-depressant use), exposure to environmental chemicals (e.g., mercury, phthalates, and polyfluoroalkyl chemicals), and maternal physical and mental health (e.g., stress, depression, obesity, and thyroid dysfunction) have been reported to increase the risk of ADHD in children. In particular, there is strong evidence supporting an association between maternal smoking exposure during pregnancy and ADHD in children [23]. However, no studies have revealed whether DNA methylation mediates the association between maternal smoking during pregnancy and childhood ADHD symptoms.

This study aimed to explore the association among prenatal smoking exposure, ADHD symptoms at preschool age, and cord blood DNA methylation using a prospective birth cohort study, the Hokkaido Study on Environment and Children’s Health. We have previously identified the CpG sites in which cord blood DNA methylation is altered by maternal smoking during pregnancy using the Illumina Infinium HumanMethylation450 BeadChips [24]. In this study, we selected the CpG sites of five genes (*AHRR*, *CYP1A1*, estrogen receptor 1 [*ESR1*], *MYO1G*, and *GFI1*) whose DNA methylation was significantly altered by maternal smoking during pregnancy; DNA methylation rates were measured using bisulfite next-generation sequencing. Next, we evaluated whether DNA methylation differences in these genes mediated the association between prenatal smoking exposure and ADHD symptoms.

## Results

Maternal cotinine levels are represented in Table 1. Of the 1150 mothers, 612 (53.2%) were categorized as non-smokers, 429 (37.3%) as passive smokers, and 109 (9.5%) as active smokers. There were 188 (16.3%) children with ADHD symptoms. Maternal age, parity, education, household income, and ADHD symptoms were significantly different among the three categories. The birth weight of infants of active smokers was lower than that of infants of non-smokers or passive smokers.

**Table 1:** Study population characteristics according to maternal cotinine levels

	All	Maternal cotinine (ng/ml)			*P* value
		Non-smokers	Passive smokers	Active smokers	
	(*n* = 1150)	(*n* = 612)	(*n* = 429)	(*n* = 109)	
*Mothers*					
Age (years)	31.3 ± 4.4	31.9 ± 4.2	30.5 ± 4.5	31.2 ± 4.6	< 0.001
BMI (kg/m^2^)	21.1 ± 3.1	21.2 ± 3.2	20.9 ± 2.8	21.5 ± 4.0	0.167
*Parity*					< 0.001
Primiparous	495 (43.0)	236 (38.6)	223 (52.0)	36 (33.0)	
Multiparous	652 (56.7)	373 (60.9)	206 (48.0)	73 (67.0)	
Missing data	3 (0.3)	3 (0.5)	0 (0.0)	0 (0.0)	
*Alcohol intake during pregnancy*					0.061
No	1016 (88.3)	551 (90.0)	375 (87.4)	90 (82.6)	
Yes	134 (11.7)	61 (10.0)	54 (12.6)	19 (17.4)	
*Education (years)*					< 0.001
≤ 12	468 (40.7)	216 (35.3)	182 (42.4)	70 (64.2)	
> 12	681 (59.2)	395 (64.5)	247 (57.6)	39 (35.8)	
Missing data	1 (0.1)	1 (0.2)	0 (0.0)	0 (0.0)	
*Annual household income (million JPY)*					0.002
5 <	743 (64.6)	368 (60.1)	295 (68.8)	80 (73.4)	
≥ 5	407 (35.4)	244 (39.9)	134 (31.2)	29 (26.6)	
Infants					
*Sex*					0.095
Male	579 (50.3)	290 (47.4)	232 (54.1)	57 (52.3)	
Female	571 (49.7)	322 (52.7)	197 (45.9)	52 (47.7)	
Birth weight (g)	3070.2 ± 366.6	3065.0 ± 358.3	3118.2 ± 353.9	2910.7 ± 415.5	< 0.001
*Gestational age (weeks)*					0.338
Preterm (< 37 weeks)	28 (2.4)	17 (2.8)	7 (1.6)	4 (3.7)	
Full-term (≥ 37 weeks)	1122 (97.6)	595 (97.2)	422 (98.4)	105 (96.3)	
*ADHD symptoms*					0.011
No	962 (83.7)	527 (86.1)	353 (82.3)	82 (75.2)	
Yes	188 (16.3)	85 (13.9)	76 (17.7)	27 (24.8)	

Data are presented as mean ± standard deviation (SD) or number (%)

*P* values derived from the Welch's test or Chi-square test

Table 2 summarizes the results of univariate and multivariate analyses on the association between maternal smoking exposure during pregnancy and child ADHD symptoms at the age of 6. Maternal active smoking during pregnancy was significantly associated with an increased risk of childhood ADHD symptoms in univariate (odds ratio [OR], 2.04; 95% confidence interval [CI], 1.25–3.34) and multivariate (OR, 1.89; 95% CI 1.14–3.15) logistic regression analyses. No significant association was observed between maternal passive smoking during pregnancy and ADHD symptoms.

**Table 2:** Association of maternal cotinine levels with ADHD symptoms

	Crude OR (95% CI)	Adjusted OR (95% CI)
Non-smokers	Reference	Reference
Passive smokers	1.33 (0.95, 1.87)	1.17 (0.82, 1.66)
Active smokers	**2.04 (1.25, 3.34)**	**1.89 (1.14, 3.15)**

Adjusted for maternal age, family income, maternal alcohol consumption during pregnancy, parity, child sex, pre-pregnancy BMI

Bold text indicates a statistically significant difference with a *p* value < 0.05

**Fig. 1:**
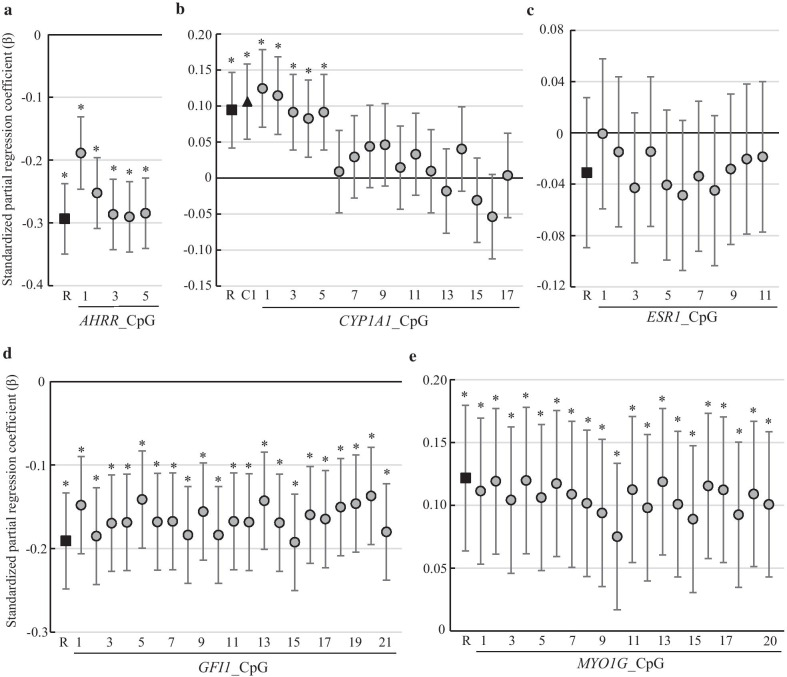
The association between maternal smoking exposure during pregnancy and DNA methylation. **a** *AHRR*, **b** *CYP1A1*, **c** *ESR1*, **d** *GFI1*, and **e** *MYO1G*. The squares indicate the average methylation of all analyzed CpG sites (regions). The triangle indicates the CpG cluster shown in Additional file 1: Fig. S1. The circles indicate the methylation of individual CpG sites. Standardized partial regression coefficient (*β*) was adjusted for maternal age, family income, maternal alcohol consumption during pregnancy, parity, child sex, and pre-pregnancy BMI. The error bars display the 95% confidence intervals. *p < 0.05

Based on our previous EWAS results, we focused on the DNA methylation of five genes (*AHRR*, *CYP1A1*, *ESR1*, *GFI1*, and *MYO1G*) associated with prenatal smoke exposure. The analyzed regions of five genes are shown in Additional file 1: Fig. S1. Since the methylation changes due to smoking exposure changed in the same direction in all CpGs (defined as a region) in each amplicon, the average methylation in the region was also used as a methylation index. We assessed the association between maternal smoking exposure during pregnancy and DNA methylation rate (Fig. 1 and Additional file 1: Table S1). Multivariate analysis revealed a significant negative association between maternal smoking exposure during pregnancy and DNA methylation rates on a region of *AHRR* (*β* =  − 0.29; 95% CI − 0.35 to − 0.24) and *GFI1* (*β* =  − 0.19; 95% CI − 0.25 to − 0.13). Meanwhile, a significant positive association was observed between maternal smoking exposure during pregnancy and DNA methylation rates on a region of *MYO1G* (*β* = 0.12; 95% CI 0.06–0.18) and *CYP1A1* (*β* = 0.09; 95% CI 0.04–0.15). However, the methylation rates of *CYP1A1* were significantly different before CpG5 (effect size: approximately 3%) and after CpG6 (effect size: approximately 0.3%) (Additional file 1: Fig. S2b). Therefore, CpG1 to CpG5 of *CYP1A1* were analyzed as cluster 1 (Additional file 1: Fig. S1b). The partial regression coefficient increased by 0.02 compared to the *CYP1A1* region (*β* = 0.11; 95% CI 0.05–0.16). No significant association was observed between maternal smoking exposure during pregnancy and DNA methylation rates both on a region and individual CpG site of *ESR1*.

**Fig. 2:**
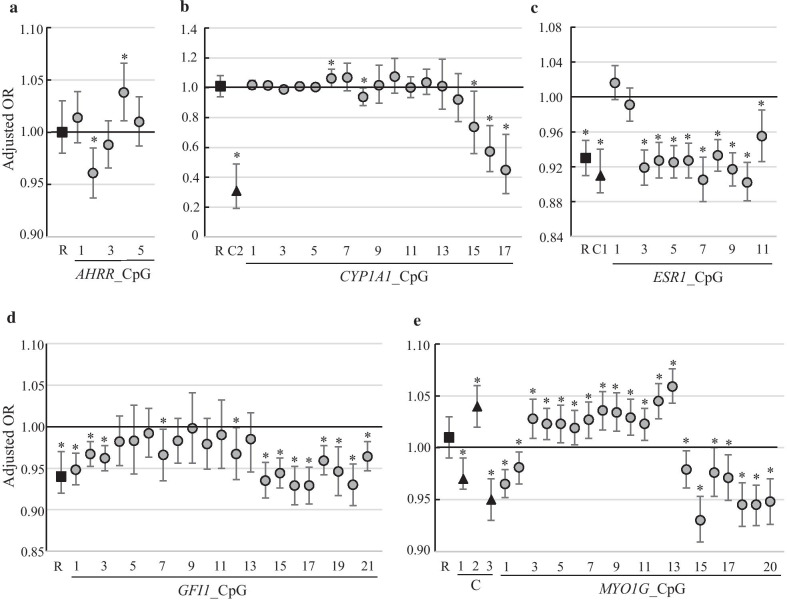
Association of DNA methylation with ADHD symptoms by logistic regression analysis. **a** *AHRR*, **b** *CYP1A1*, **c** *ESR1*, **d** *GFI1*, and **e** *MYO1G*. Odds ratios were adjusted for maternal age, family income, maternal cotinine levels, maternal alcohol consumption during pregnancy, parity, child sex, and pre-pregnancy BMI. The squares indicate the average methylation of all analyzed CpG sites (regions). The triangle indicates the CpG cluster shown in Fig. S1. The circles indicate the methylation of individual CpG sites. The error bars display the 95% confidence intervals. *p < 0.05

Next, we examined the association between DNA methylation and childhood ADHD symptoms using logistic regression analysis. Figure 2 and Additional file 1: Table S2 show the ORs and their 95% CIs for the risk of ADHD symptoms in relation to DNA methylation. After adjustment for potential covariates, including maternal smoking exposure during pregnancy, a one-unit percent (%) increase in DNA methylation rates on a region of *ESR1* (OR, 0.93; 95% CI 0.91–0.95) and *GFI1* (OR, 0.94; 95% CI 0.92–0.97) regions was associated with significantly lower odds of ADHD symptoms. No significant association was observed between DNA methylation rates on *AHRR*, *CYP1A1*, and *MYO1G* and childhood ADHD symptoms. Based on the results of the association between individual CpG methylation and ADHD, we defined CpG clusters in *CYP1A1* (Additional file 1: Fig. S1b), *ESR1* (Additional file 1: Fig. S1c), and *MYO1G* (Additional file 1: Fig. S1e). When analyzed at a cluster level, a one-unit increase (%) in the DNA methylation of *CYP1A1* cluster 2 (OR, 0.31; 95% CI 0.19–0.49), *ESR1* cluster 1 (OR, 0.91; 95% CI 0.89–0.94), and *MYO1G* clusters 1 and 3 (OR, 0.97; 95% CI 0.96–0.99 and OR, 0.95; 95% CI 0.93–0.97, respectively) was associated with significantly lower odds of ADHD symptoms. Meanwhile, a one-unit increase (%) in the DNA methylation of *MYO1G* cluster 2 (OR, 1.04; 95% CI 1.02–1.06) was associated with significantly higher odds of ADHD symptoms.

**Fig. 3:**
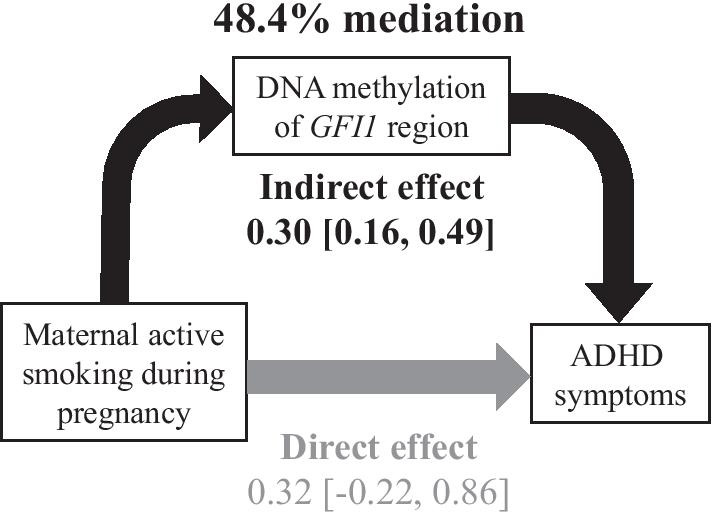
Mediation of the association between active smoking during pregnancy and ADHD symptoms via DNA methylation of *GFI1* regions. Mediation analysis was adjusted for maternal alcohol consumption during pregnancy, family income, pre-pregnancy BMI, parity, gestational age, and infant sex. Percent mediation was calculated as the indirect effect derived by the total (indirect + direct) effect × 100%

To elucidate whether DNA methylation changes mediated the association between prenatal smoke exposure and ADHD symptoms, a mediation analysis was performed. The results indicated that DNA methylation of the *GFI1* region mediated 48.4% of the total effect of the association between maternal active smoking during pregnancy and ADHD symptoms (Fig. 3). DNA methylation of other genes regions did not exert a statistically significant mediation effect (Additional file 1: Table S3).

## Discussion

The molecular mechanisms linking maternal smoking during pregnancy to childhood ADHD symptoms remain unclear. This is the first study showing that maternal active smoking during pregnancy may be associated with ADHD symptoms at preschool age through DNA methylation of *GFI1*.

In a previous study, using the Strengths and Difficulties Questionnaire (SDQ), we reported that maternal active smoking during pregnancy was significantly associated with an increased risk of total difficulties and hyperactivity/inattention in 5-year-old children [25]. This study also revealed that maternal active smoking during pregnancy was significantly associated with an increased risk of ADHD in their children, based on the Attention-Deficit Hyperactivity Disorder-Rating Scale (ADHD-RS) at the age of 6 years. In contrast, the impact of passive smoking is controversial. In this study, no significant association was found between passive smoking during pregnancy and ADHD. Several epidemiological studies suggest that the association between pregnant mother smoking and ADHD is due to various confounding factors such as parental psychiatric history and social factors [26–28]. Our findings were consistent with those of previous studies showing that maternal smoking during pregnancy was associated with ADHD after adjusting for socioeconomic status. However, ADHD is characterized by a high inheritability (> 70%) [29], but this study did not consider the parents’ history of ADHD. Furthermore, it has been suggested that postnatal factors such as breastfeeding [30], second-hand smoke exposure [31], and maternal postnatal mental health [32] are associated with ADHD in children, but this study has not considered them.

In our previous study, we used a different cohort "Hokkaido Study Sapporo Cohort" to identify methylation site changes in cord blood due to maternal smoking exposure during pregnancy by a 450 K array and verified *AHRR*_ cg05575921 and *CYP1A1*_ cg05549655 by bisulfite sequence using NGS [24]. The five CpGs of *AHRR* in this study correspond to exactly the same sequence as that used in our previous report. The five CpGs of *CYP1A1* analyzed in our previous study correspond to CpG1 to CpG5 in this study (Additional file 1: Fig. S1b). With regard to these CpGs, similar methylation changes were confirmed in this study for all CpGs except *CYP1A1*_CpG4 (Additional file 1: Fig. S2b). In this study, the CpGs of *GFI1* (cg12876356 and cg18146737) and *MYO1G* (cg12803068 and cg04180046) identified from the 450 K were confirmed to have similar methylation patterns in the NGS analysis. However, the CpGs of *ESR1* (cg04063345 and cg15626350) and *CYP1A1* (cg23727072 and cg00213123) did not match with the previous results. The possible reasons for this discrepancy are as follows: (1) Differences in methylation analysis methods (450 K array vs. bisulfite sequencing); (2) Differences in sample sizes (previous: 247, current: 1150); (3) Different groupings of maternal smoking exposure (previous: questionnaire, current: cotinine concentration); (4) Possible false positives in the 450 K analysis.

Wilmot et al. first reported the EWAS study which identified the genes with altered DNA methylation associated with ADHD using saliva samples, such as *MYT1L* and *VIPR*2 [18]. Furthermore, a large integrated genetic/epigenetic study using saliva samples reported an association between ADHD and DNA methylation at several novel CpG sites such as *SLC7A8*, *MARK2*, and *SON* [19]. A prospective study, the Avon Longitudinal Study of Parents and Children (ALSPAC), based on the EWAS approach identified 13 genes where the level of DNA methylation at birth and at age 7 years was significantly associated with differentiated ADHD trajectories between 7 and 15 years of age [21]. This study revealed that an increase in DNA methylation of *ESR1* cluster 1 at birth was associated with significantly lower odds of ADHD symptoms at age 6 regardless of smoking exposure. ESR1, one of two ESR subtypes, is a nuclear receptor that is activated by the sex hormone estrogen. Single nucleotide polymorphisms within the *ESR1* gene are associated with neuropsychiatric disorders including ADHD [33, 34]. According to animal studies, prenatal exposure to chemicals such as phthalates and benzophenone-3 impairs neurodevelopment via disruption of *Esr* expression and alteration of the epigenetic status [35, 36]. Our results suggest that DNA methylation of *ESR1* may be a novel potential biomarker of ADHD symptoms. Increased DNA methylation of *CYP1A1* cluster 2 at birth was also associated with significantly lower odds of ADHD symptoms at age 6. Since DNA methylation rates are as low as less than 1% (Additional file 1: Fig. S2b), further verification is needed to determine if slight methylation changes in *CYP1A1* are associated with ADHD symptoms.

This study showed that hypomethylation at *GFI1* in umbilical cord blood could explain the positive association between maternal active smoking during pregnancy and childhood ADHD symptoms. In a study on children with ADHD, Sengapta et al. reported that maternal smoking exposure during pregnancy is associated with differences in *GFI1* hypomethylation in childhood [37]. Hypomethylation at *GFI1* mediates the effect of maternal smoking exposure during pregnancy on lower birth weight [15]. These findings suggest that maternal active smoking during pregnancy may cause hypomethylation at *GFI1* and contribute to both low birth weight and ADHD symptoms during childhood. However, in this study, low birth weight did not significantly mediate the association between maternal smoking during pregnancy and childhood ADHD symptoms (data not shown). *GFI1* is a transcriptional repressor that plays an important role in diverse developmental contexts such as hematopoiesis and oncogenesis. GFI1 is involved in the regulation of the T helper type 1 (Th1)-type immune response as well as the promotion of T helper type 2 (Th2) cell development [38]. ADHD has high comorbidity with both Th1- and Th2-mediated disorders such as ear infections and atopic diseases [39]. There is indeed strong evidence that ADHD is associated with atopic diseases and that individuals suffering from atopic diseases have a 30–50% greater chance of developing ADHD [40]. However, it is not clear how *GFI1* is involved in the molecular mechanisms of ADHD.

DNA methylation analysis by bisulfite sequence using a next-generation sequencer can clarify the methylation state of CpG around the probe of the methylation array. In addition to the methylation analysis of individual CpGs, we analyzed the average methylation of all CpGs contained in the amplicon (defined as a region). It is also important to consider smoking- or ADHD-associated differentially methylated regions (DMR). The association among *CYP1A1*, *ESR1*, and *MYO1G* methylation and ADHD symptoms is clearly clustered within the region (Fig. 2b, c and e). In particular, DNA methylation in the *MYO1G* region showed no statistically significant mediating effect, but hypermethylation of *MYO1G* cluster 2 explained the positive association (28.1% of the total effect) between active smoking during pregnancy and ADHD symptoms. In contrast, hypomethylation of *MYO1G* clusters 1 and 3 only partially explained the negative association between active smoking during pregnancy and ADHD symptoms. These results reveal that hypermethylation of *MYO1G* in active smokers during pregnancy is involved in both increased and weakened risk of ADHD symptoms. However, the amplicons in this study were arbitrarily designed to include methylation sites associated with maternal smoking exposure during pregnancy from a previous EWAS study. Therefore, the research is limited in that the interpretation of methylation in regions and clusters in this study may change depending on the methylation state around the amplicon.

It is well known that DNA methylation of promoter sequences acts to repress gene transcription. In contrast, gene-body DNA methylation has been reported to be associated with both gene activation and suppression [41, 42]. *MYO1G* regions analyzed in this study are located in a CpG island and exon 21 near the 3′ gene region. Hypermethylation in this gene region might correlate with active transcription of *MYO1G* [43, 44]. The region of *GFI1* analyzed in this study is located in intron 3 and exon 4 (Additional file 1: Fig. S1d), and its effect on gene expression remains unknown. Therefore, gene-body DNA methylation associated with ADHD should be examined together with gene expression levels.

Adverse environmental conditions during the fetal period to early childhood are linked to an increased risk of non-communicable diseases in adulthood. This concept is called DOHaD. Epigenetic modifications, such as DNA methylation, histone modification, and non-coding RNA, are thought to be molecular mechanisms of DOHaD. A limited number of studies have reported on outcomes other than birth weight. Parmar et al*.* [45] reported that DNA methylation at the *GFI1* locus is associated with maternal prenatal smoking and cardiometabolic risk factors. Furthermore, long-term prenatal exposure to paracetamol (acetaminophen) is associated with DNA methylation differences in children diagnosed with ADHD [22]. To the best of our knowledge, this is the first study to clarify the association between prenatal smoking exposure and ADHD risk in children from the viewpoint of the molecular mechanism of DOHaD.

This study has several limitations. First, ADHD suspected symptoms were not diagnosed but screened by ADHD-RS questionnaire; hence, there is a possibility of misclassification. However, previous studies have confirmed the reliability and validity of ADHD-RS for screening children in Japan [46, 47]. Second, umbilical cord blood-derived DNA methylation does not consider differences in cell composition. The EWAS applied several (reference-based and reference-free) methods for adjusting a difference in blood cell composition using a 450 K and EPIC array [48, 49]. However, these methods are not directly applicable to candidate locus-specific DNA methylation analyses [37]. Smoking exposure can affect the cell type composition of blood. However, it has been reported that the difference in methylation due to smoking is greater than the difference in methylation between the two major pools of mononuclear cells (mainly lymphocytes) and granulocytes (mainly polymorphonuclear leukocytes) [50]. It is suggested that confounding by cell type is unlikely to rule out the results of smoking-related methylation differences.

Finally, DNA methylation patterns differ between tissues and cell types. We do not know whether the methylation changes in cord blood DNA also occur in brain tissue DNA. However, correlation of DNA methylation between blood and brain, and association between blood DNA methylation and brain phenotypes have been reported [51, 52]. Thus, DNA methylation in cord blood may serve as an indicator of neurodevelopmental disorders.

## Conclusions

Our findings, taken together, have demonstrated that maternal active smoking during pregnancy was associated with altered DNA methylation and ADHD symptoms in children of preschool age. DNA methylation of *GFI1* mediated the effect of maternal active smoking on ADHD symptoms.

## Methods

### Study participants and baseline survey

This prospective birth cohort study was a part of the Hokkaido Study on the Environment and Children’s Health. The study design and procedures have been described previously in detail [53, 54]. Among all participants of 20,926 pregnant women who registered to the study from 2002 to 2012, this study comprised 3817 pregnant women and their offspring, delivered between March 2008 and April 2010. Pregnant women completed the baseline questionnaire, and peripheral blood sampling was conducted with prenatal health checkup by obstetricians; medical records and cord blood were taken at delivery. The questionnaire included maternal information about age, height, weight before pregnancy, parity, alcohol consumption during the first trimester, and annual household income. Birth weight, gestational age, and infant sex were obtained from medical records. A total of 1150 mother–infant pairs were finally included (Additional file 1: Fig. S3). The child's mother mainly filled out the questionnaire, including the ADHD survey for the child. In a few cases, when the mother withdrew from the study, the father or other guardian took over the responsibility to fill out the questionnaire. This study was conducted with informed consent of all subjects in writing. All procedures involving human subjects were approved by the University of Yamanashi, the Hokkaido University Graduate School of Medicine, and the Hokkaido University Center for Environmental and Health Science and were performed in accordance with relevant guidelines and regulations.

### Cotinine level measurement

Plasma cotinine levels at the third trimester of pregnancy were measured using a highly sensitive enzyme-linked immunosorbent assay kit (Cosmic Corp., Tokyo, Japan). The detailed protocol has been described previously [55]. Based on our previous reports, which determined the cut-off levels for cotinine in non-smokers (≤ 0.21 ng/mL), passive smokers (0.21–11.48 ng/mL), and active smokers (≥ 11.48 ng/mL), we categorized the mothers into three groups.

### Follow-up and outcome assessment

The ADHD-RS IV (home version) [56] was designed for assessment of two features of ADHD: inattention (nine items) and hyperactivity-impulsivity (nine items). We shared the Japanese version of the ADHD-RS IV via mail with 2735 participants and the parents completed the questionnaire to assess their child at the age of 6. From the 1368 questionnaire responses collected until May 2016 (response rate 50.0%), we calculated the total score by adding the scores of the inattention and hyperactivity-impulsivity scales. Tanaka concluded the Japanese version of the ADHD-RS IV shows good reliability and validity for screening children with possible ADHD [47]. We adopted Tanaka’s cut-off criteria, namely, the 80^th^ percentile score for 5–7 yeas children (14 point for boys and 9.4 point for girls, respectively), to extract suspect-ADHD children.

### DNA methylation analysis

Umbilical cord blood samples were acquired immediately after birth and stored at − 80 °C. Genomic DNA was extracted from cord blood using a Maxwell® 16 DNA Purification Kit (Promega, Madison, WI, USA). DNA was subjected to bisulfite conversion by using an EZ DNA Methylation-Lightning Kit (Zymo Research, Irvine, CA, USA). Bisulfite-treated DNA was then amplified using FastStart Taq DNA Polymerase (Roche, Basel, Schweiz). Polymerase chain reaction (PCR) primers for bisulfite PCR were designed using MethPrimer (Additional file 1: Table S4). For next-generation sequencing, amplicon libraries were generated using an Ion Plus Fragment Library Kit (Thermo Fisher Scientific, MA, USA) as described previously [24]. Sequencing was performed using an Ion PGM Hi-Q View Sequencing Kit and 850 flows on an Ion 318 Chip Kit v2 (Thermo Fisher Scientific). After sequencing, single processing and base calling were performed using Torrent Suite 5.12.1 (Thermo Fisher Scientific). Methylation analysis was performed using Methylation Analysis_Amplicon plug-in v2.1 (Thermo Fisher Scientific). The analyzed regions of five genes are shown in Fig. 1. The number of CpG contained in the amplicon was 5 for *AHRR*, 17 for *CYP1A1*, 11 for *ESR1*, 21 for *GFI*, and 20 for *MYO1G*. The methylation rates of individual CpGs were used as a percentage. The methylation rates of each gene region were obtained by calculating the average of all CpGs in the amplicon. CpG clusters were defined as follows: in CYP1A1, CpG1 to CpG5 formed cluster 1 and CpG15 to CpG17 cluster 2 (Fig. 1b). In ESR, CpG3 to CpG11 were cluster 1 (Fig. 1c). In MYO1G, CpG1 and CpG2 were cluster 1, CpG3 to CpG13 cluster 2, and CpG14 to CpG20 cluster 3 (Fig. 1e). The methylation rates of each cluster were obtained by calculating the average of all CpGs in the cluster.

### Statistical analysis

Based on a literature review of observational studies, maternal alcohol consumption during pregnancy, family income, pre-pregnancy body mass index (BMI), parity, gestational age, and infant sex were selected as possible confounders [8, 57]. The association between maternal smoking during pregnancy and ADHD symptoms in children was examined using a logistic regression analysis adjusted for possible confounders. The association between maternal smoking during pregnancy and DNA methylation was examined by multiple linear regression analysis adjusted for possible confounders. The association between DNA methylation and ADHD symptoms in children was examined using logistic regression analysis adjusted for possible confounders and maternal smoking exposure during pregnancy.

Finally, a mediation analysis was used to estimate the degree of the association between prenatal smoking exposure and ADHD symptoms, which is explained by DNA methylation changes. The direct effect was the effect of the exposure (X) on the outcome (Y) at a fixed level of the mediator (M). The indirect effect of X on Y through M can be quantified as the product of two coefficients: a (the effect of X on M) and b (the effect of M on Y) pathways (i.e., ab). Percent mediation was calculated as the indirect effect divided by the total (indirect + direct) effect × 100%. Mediation analysis included maternal alcohol consumption during pregnancy, family income, pre-pregnancy BMI, parity, gestational age, and infant sex as covariates. The bias-corrected and accelerated CIs of the indirect effect (ab) were calculated by bootstrapping with 5000 iterations [58]. Mediation analysis was performed by using PROCESS version 3.5, a macro implemented in SPSS (IBM, Armonk, NY, USA). All statistical analyses were performed using SPSS version 27. A p-value of 0.05 (two-sided) was considered statistically significant.

## Supplementary Information

**Additional file 1: Fig. S1.** Base sequences analyzed by targeted bisulfite next-generation sequencing. **Fig. S2.** Comparison of methylated CpG sites among non-smokers, passive smokers, and active smokers. **Fig. S3.** Selection of the study population. **Table S1.** Association between maternal smoking during pregnancy and umbilical cord blood DNA methylation. **Table S2.** Association of umbilical cord blood DNA methylation with ADHD symptoms at 6 years of age. **Table S3.** Mediation analysis for the effect of DNA methylation in the association between active smoking during pregnancy and ADHD symptoms at 6 years of age. **Table S4.** List of bisulfite PCR primers.

## Data Availability

The datasets used and analyzed during the present study are available from the corresponding author on reasonable request.

## Abbreviations

ADHD: Attention-deficit/hyperactivity disorder
ADHD-RS: Attention-Deficit Hyperactivity Disorder-Rating Scale
AHRR: Aryl-hydrocarbon receptor repressor
BMI: Body mass index
CI: Confidence interval
CYP1A1: Cytochrome P450 family 1 subfamily A member 1
DOHaD: Developmental Origins of Health and Disease
ESR1: Estrogen receptor 1
EWAS: Epigenome-wide association study
GFI1: Growth factor-independent 1 transcriptional repressor
MYO1G: Myosin IG
OR: Odds ratio
PCR: Polymerase chain reaction
SDQ: Strengths and Difficulties Questionnaire
Th1: T helper type 1
Th2: T helper type 2
