# Supplementary material for: DNA methylation of GFI1 as a mediator of the association between prenatal smoking exposure and ADHD symptoms at 6 years: the Hokkaido Study on Environment and Children’s Health
Source: Clin Epigenetics. 2021 Apr 7;13:74. doi: 10.1186/s13148-021-01063-z (PMC8028116; doi:10.1186/s13148-021-01063-z)
Supplement: Supplementary file 1 — Additional file 1: Fig. S1. Base sequences analyzed by targeted bisulfite next-generation sequencing. Fig. S2. Comparison of methylated CpG sites among non-smokers, passive smokers, and active smokers. Fig. S3. Selection of the study population. Table S1. Association between maternal smoking during pregnancy and umbilical cord blood DNA methylation. Table S2. Association of umbilical cord blood DNA methylation with ADHD symptoms at 6 years of age. Table S3. Mediation analysis for the effect of DNA methylation in the association between active smoking during pregnancy and ADHD symptoms at 6 years of age. Table S4. List of bisulfite PCR primers. [file 13148_2021_1063_MOESM1_ESM.docx]

**Additional file 1: Fig. S1.** **Base sequences analyzed by targeted bisulfite next-generation sequencing.** CpG sites are indicated by serial numbers and underlines. CpG sites shown in red are the CpG sites that changed with prenatal smoking exposure, as identified by EWAS. The amplified sequence of each gene was defined as a region. (b) CpG sites 1 to 5 and 15 to 17 of CYP1A1 were defined as clusters 1 and 2, respectively. (c) CpG sites 3 to 11 of ESR1 were defined as cluster 1. (e) CpG sites 1 to 2, 3 to 13, and 14 to 20 of MYO1G were defined as clusters 1, 2, and 3, respectively.

**Additional file 1: Fig. S2.** **Comparison of methylated CpG sites among non-smokers, passive smokers, and active smokers.** (a) *AHRR*, (b) *CYP1A1*, (c) *ESR1*, (d) *GFI1*, and (e) *MYO1G*. Methylation levels (%) at each CpG and average of all CpGs (R) in each amplicon are indicated for each smoking category. The error bars display the 95% confidence intervals. The DNA methylation rates of region (R), cluster (C1) and each CpG among 3 groups were analyzed by the Bonferroni correction for the number of CpGs tested. *P < 0.05, **P < 0.01.

**Additional file 1: Fig. S3. Selection of the study population.**

**Additional file 1: Table S1. Association between maternal smoking exposure during pregnancy and umbilical cord blood DNA methylation.**

|  | **Unadjusted** | | **Adjusted** | |
| --- | --- | --- | --- | --- |
|  | **β** | **95 % CI** | **β** | **95 % CI** |
| AHRR_region | **-0.29** | **(-0.35, -0.24)** | **-0.29** | **(-0.35, -0.24)** |
| AHRR_CpG1 | **-0.19** | **(-0.25, -0.13)** | **-0.19** | **(-0.25, -0.13)** |
| AHRR_CpG2 | **-0.25** | **(-0.31, -0.20)** | **-0.25** | **(-0.31, -0.20)** |
| AHRR_CpG3 | **-0.29** | **(-0.34, -0.23)** | **-0.29** | **(-0.34, -0.23)** |
| AHRR_CpG4 | **-0.29** | **(-0.35, -0.23)** | **-0.29** | **(-0.35, -0.23)** |
| AHRR_CpG5 | **-0.28** | **(-0.34, -0.23)** | **-0.28** | **(-0.34, -0.23)** |
| CYP1A1_region | **0.07** | **(0.02, 0.13)** | **0.09** | **(0.04, 0.15)** |
| CYP1A1_cluster1 | **0.08** | **(0.03, 0.14)** | **0.11** | **(0.05, 0.16)** |
| CYP1A1_CpG1 | **0.11** | **(0.05, 0.16)** | **0.12** | **(0.07, 0.18)** |
| CYP1A1_CpG2 | **0.10** | **(0.04, 0.15)** | **0.11** | **(0.06, 0.17)** |
| CYP1A1_CpG3 | **0.07** | **(0.01, 0.13)** | **0.09** | **(0.04, 0.14)** |
| CYP1A1_CpG4 | **0.06** | **(0.00, 0.12)** | **0.08** | **(0.03, 0.14)** |
| CYP1A1_CpG5 | **0.07** | **(0.01, 0.13)** | **0.09** | **(0.04, 0.14)** |
| CYP1A1_CpG6 | 0.00 | (-0.06, 0.06) | 0.01 | (-0.05, 0.07) |
| CYP1A1_CpG7 | 0.03 | (-0.03, 0.08) | 0.03 | (-0.03, 0.09) |
| CYP1A1_CpG8 | 0.04 | (-0.02, 0.09) | 0.04 | (-0.01, 0.10) |
| CYP1A1_CpG9 | 0.03 | (-0.03, 0.09) | 0.05 | (-0.01, 0.10) |
| CYP1A1_CpG10 | 0.00 | (-0.05, 0.06) | 0.01 | (-0.04, 0.07) |
| CYP1A1_CpG11 | 0.02 | (-0.04, 0.07) | 0.03 | (-0.02, 0.09) |
| CYP1A1_ CpG 12 | 0.00 | (-0.06, 0.06) | 0.01 | (-0.05, 0.07) |
| CYP1A1_ CpG 13 | -0.02 | (-0.08, 0.04) | -0.02 | (-0.08, 0.04) |
| CYP1A1_ CpG 14 | 0.04 | (-0.06, 0.06) | 0.04 | (-0.02, 0.10) |
| CYP1A1_ CpG 15 | -0.03 | (-0.08, 0.04) | -0.03 | (-0.09, 0.03) |
| CYP1A1_ CpG 16 | -0.05 | (-0.11, 0.01) | -0.05 | (-0.11, 0.01) |
| CYP1A1_ CpG 17 | 0.00 | (-0.06, 0.05) | 0.00 | (-0.06, 0.06) |
| ESR1_region | -0.03 | (-0.09, 0.03) | -0.03 | (-0.09, 0.03) |
| ESR1_ CpG 1 | 0.00 | (-0.05, 0.06) | 0.00 | (-0.06, 0.06) |
| ESR1_ CpG 2 | -0.01 | (-0.07, 0.05) | -0.01 | (-0.07, 0.04) |
| ESR1_ CpG 3 | -0.04 | (-0.10, 0.01) | -0.04 | (-0.10, 0.02) |
| ESR1_ CpG 4 | -0.02 | (-0.07, 0.04) | -0.01 | (-0.07, 0.04) |
| ESR1_ CpG 5 | -0.04 | (-0.10, 0.02) | -0.04 | (-0.10, 0.02) |
| ESR1_ CpG 6 | -0.05 | (-0.11, 0.01) | -0.05 | (-0.11, 0.01) |
| ESR1_ CpG 7 | -0.03 | (-0.09, 0.02) | -0.03 | (-0.09, 0.02) |
| ESR1_ CpG 8 | -0.05 | (-0.11, 0.01) | -0.04 | (-0.10, 0.01) |
| ESR1_ CpG 9 | -0.03 | (-0.09, 0.02) | -0.03 | (-0.09, 0.03) |
| ESR1_ CpG 10 | -0.02 | (-0.08, 0.04) | -0.02 | (-0.08, 0.04) |
| ESR1_ CpG 11 | -0.02 | (-0.07, 0.04) | -0.02 | (-0.08, 0.04) |
| GFI1_region | **-0.18** | **(-0.24, -0.12)** | **-0.19** | **(-0.25, -0.13)** |
| GFI1_ CpG 1 | **-0.15** | **(-0.20, -0.09)** | **-0.15** | **(-0.21, -0.09)** |
| GFI1_ CpG 2 | **-0.17** | **(-0.23, -0.12)** | **-0.19** | **(-0.24, -0.13)** |
| GFI1_ CpG 3 | **-0.16** | **(-0.22, -0.10)** | **-0.17** | **(-0.23, -0.11)** |
| GFI1_ CpG 4 | **-0.16** | **(-0.22, -0.10)** | **-0.17** | **(-0.23, -0.11)** |
| GFI1_ CpG 5 | **-0.13** | **(-0.19, -0.07)** | **-0.14** | **(-0.20, -0.08)** |
| GFI1_ CpG 6 | **-0.16** | **(-0.22, -0.10)** | **-0.17** | **(-0.23, -0.11)** |
| GFI1_ CpG 7 | **-0.16** | **(-0.21, -0.10)** | **-0.17** | **(-0.23, -0.11)** |
| GFI1_ CpG 8 | **-0.17** | **(-0.23, -0.11)** | **-0.18** | **(-0.24, -0.13)** |
| GFI1_ CpG 9 | **-0.15** | **(-0.20, -0.09)** | **-0.16** | **(-0.21, -0.10)** |
| GFI1_ CpG 10 | **-0.17** | **(-0.23, -0.11)** | **-0.18** | **(-0.24, -0.13)** |
| GFI1_ CpG 11 | **-0.16** | **(-0.21, -0.10)** | **-0.17** | **(-0.23, -0.11)** |
| GFI1_ CpG 12 | **-0.16** | **(-0.22, -0.10)** | **-0.17** | **(-0.23, -0.11)** |
| GFI1_ CpG 13 | **-0.13** | **(-0.19, -0.07)** | **-0.14** | **(-0.20, -0.08)** |
| GFI1_ CpG 14 | **-0.16** | **(-0.22, -0.11)** | **-0.17** | **(-0.23, -0.11)** |
| GFI1_ CpG 15 | **-0.19** | **(-0.24, -0.13)** | **-0.19** | **(-0.25, -0.13)** |
| GFI1_ CpG 16 | **-0.16** | **(-0.21, -0.10)** | **-0.16** | **(-0.22, -0.10)** |
| GFI1_ CpG 17 | **-0.16** | **(-0.22, -0.10)** | **-0.16** | **(-0.22, -0.11)** |
| GFI1_ CpG 18 | **-0.15** | **(-0.20, -0.09)** | **-0.15** | **(-0.21, -0.09)** |
| GFI1_ CpG 19 | **-0.14** | **(-0.20, -0.08)** | **-0.15** | **(-0.20, -0.09)** |
| GFI1_ CpG 20 | **-0.13** | **(-0.18, -0.08)** | **-0.14** | **(-0.20, -0.08)** |
| GFI1_ CpG 21 | **-0.17** | **(-0.23, -0.12)** | **-0.18** | **(-0.24, -0.12)** |
| MYO1G_region | **0.11** | **(0.06, 0.17)** | **0.12** | **(0.06, 0.18)** |
| MYO1G_ CpG 1 | **0.10** | **(0.05, 0.16)** | **0.11** | **(0.05, 0.17)** |
| MYO1G_ CpG 2 | **0.11** | **(0.05, 0.17)** | **0.12** | **(0.06, 0.18)** |
| MYO1G_ CpG 3 | **0.10** | **(0.04, 0.15)** | **0.10** | **(0.05, 0.16)** |
| MYO1G_ CpG 4 | **0.11** | **(0.05, 0.17)** | **0.12** | **(0.06, 0.18)** |
| MYO1G_ CpG 5 | **0.10** | **(0.04, 0.16)** | **0.11** | **(0.05, 0.16)** |
| MYO1G_ CpG 6 | **0.11** | **(0.05, 0.17)** | **0.12** | **(0.06, 0.18)** |
| MYO1G_ CpG 7 | **0.10** | **(0.04, 0.16)** | **0.11** | **(0.05, 0.17)** |
| MYO1G_ CpG 8 | **0.09** | **(0.04, 0.15)** | **0.10** | **(0.04, 0.16)** |
| MYO1G_ CpG 9 | **0.08** | **(0.03, 0.14)** | **0.09** | **(0.04, 0.15)** |
| MYO1G_ CpG 10 | **0.07** | **(0.01, 0.13)** | **0.07** | **(0.02, 0.13)** |
| MYO1G_ CpG 11 | **0.11** | **(0.05, 0.16)** | **0.11** | **(0.05, 0.17)** |
| MYO1G_ CpG 12 | **0.09** | **(0.03, 0.15)** | **0.10** | **(0.04, 0.16)** |
| MYO1G_ CpG 13 | **0.11** | **(0.06, 0.17)** | **0.12** | **(0.06, 0.18)** |
| MYO1G_ CpG 14 | **0.09** | **(0.04, 0.15)** | **0.10** | **(0.04, 0.16)** |
| MYO1G_ CpG 15 | **0.09** | **(0.03, 0.14)** | **0.09** | **(0.03, 0.15)** |
| MYO1G_ CpG 16 | **0.11** | **(0.05, 0.16)** | **0.12** | **(0.06, 0.17)** |
| MYO1G_ CpG 17 | **0.10** | **(0.04, 0.16)** | **0.11** | **(0.05, 0.17)** |
| MYO1G_ CpG 18 | **0.08** | **(0.03, 0.14)** | **0.09** | **(0.03, 0.15)** |
| MYO1G_ CpG 19 | **0.10** | **(0.04, 0.16)** | **0.11** | **(0.05, 0.17)** |
| MYO1G_ CpG 20 | **0.09** | **(0.03, 0.15)** | **0.10** | **(0.04, 0.16)** |

Bold text indicates a statistically significant difference with a p-value < 0.05.

Adjusted for maternal age, family income, matrnal alcohol consumption during pregnancy, parity, child sex, pre-pregnancy BMI

**Additional file 1: Table S2. Association of umbilical cord blood DNA methylation with ADHD symptoms at 6 years of age.**

|  | **Unadjusted** | | **Adjusted** | |
| --- | --- | --- | --- | --- |
|  | **OR** | **95 % CI** | **OR** | **95 % CI** |
| AHRR_region | 0.99 | (0.96, 1.01) | 1.00 | (0.98, 1.03) |
| AHRR_CpG1 | 1.00 | (0.90, 1.03) | 1.01 | (0.99, 1.04) |
| AHRR_CpG2 | **0.95** | **(0.93, 0.98)** | **0.96** | **(0.94, 0.99)** |
| AHRR_CpG3 | **0.98** | **(0.96, 1.00)** | 0.99 | (0.97, 1.01) |
| AHRR_CpG4 | 1.01 | (0.99, 1.04) | **1.04** | **(1.01, 1.07)** |
| AHRR_CpG5 | 0.99 | (0.97, 1.02) | 1.01 | (0.99, 1.03) |
| CYP1A1_region | 1.01 | (0.95, 1.07) | 1.01 | (0.94, 1.08) |
| CYP1A1_cluster2 | **0.31** | **(0.19, 0.48)** | **0.31** | **(0.19, 0.49)** |
| CYP1A1_CpG1 | 1.02 | (0.99, 1.04) | 1.02 | (0.99, 1.05) |
| CYP1A1_CpG2 | 1.01 | (0.99, 1.04) | 1.02 | (0.99, 1.04) |
| CYP1A1_CpG3 | 0.99 | (0.97, 1.01) | 0.99 | (0.97, 1.01) |
| CYP1A1_CpG4 | 1.01 | (0.99, 1.03) | 1.01 | (0.99, 1.03) |
| CYP1A1_CpG5 | 1.00 | (0.99, 1.02) | 1.00 | (0.98, 1.03) |
| CYP1A1_CpG6 | 1.05 | (1.00, 1.11) | **1.06** | **(1.00, 1.12)** |
| CYP1A1_CpG7 | 1.05 | (0.97, 1.14) | 1.07 | (0.98, 1.16) |
| CYP1A1_CpG8 | **0.94** | **(0.89, 1.00)** | **0.94** | **(0.88, 1.00)** |
| CYP1A1_CpG9 | 1.02 | (0.90, 1.14) | 1.02 | (0.90, 1.15) |
| CYP1A1_CpG10 | 1.06 | (0.96, 1.18) | 1.07 | (0.96, 1.20) |
| CYP1A1_CpG11 | 1.00 | (0.93, 1.07) | 1.00 | (0.93, 1.07) |
| CYP1A1_ CpG 12 | 1.03 | (0.95, 1.11) | 1.04 | (0.95, 1.12) |
| CYP1A1_ CpG 13 | 1.02 | (0.87, 1.19) | 1.01 | (0.86, 1.19) |
| CYP1A1_ CpG 14 | 0.91 | (0.77, 1.09) | 0.92 | (0.77, 1.09) |
| CYP1A1_ CpG 15 | **0.71** | **(0.54, 0.93)** | **0.74** | **(0.56, 0.98)** |
| CYP1A1_ CpG 16 | **0.57** | **(0.44, 0.75)** | **0.57** | **(0.44, 0.75)** |
| CYP1A1_ CpG 17 | **0.45** | **(0.29, 0.68)** | **0.45** | **(0.29, 0.69)** |
| ESR1_region | **0.93** | **(0.91, 0.96)** | **0.93** | **(0.91, 0.95)** |
| ESR1_cluster1 | **0.92** | **(0.90, 0.94)** | **0.91** | **(0.89, 0.94)** |
| ESR1_ CpG 1 | **1.02** | **(1.00, 1.04)** | 1.02 | (1.00 1.04) |
| ESR1_ CpG 2 | 1.00 | (0.98, 1.01) | 0.99 | (0.97, 1.01) |
| ESR1_ CpG 3 | **0.92** | **(0.90, 0.94)** | **0.92** | **(0.90, 0.94)** |
| ESR1_ CpG 4 | **0.93** | **(0.91, 0.95)** | **0.93** | **(0.91, 0.95)** |
| ESR1_ CpG 5 | **0.93** | **(0.91, 0.95)** | **0.93** | **(0.91, 0.94)** |
| ESR1_ CpG 6 | **0.93** | **(0.91, 0.95)** | **0.93** | **(0.91, 0.95)** |
| ESR1_ CpG 7 | **0.91** | **(0.89, 0.94)** | **0.91** | **(0.88, 0.93)** |
| ESR1_ CpG 8 | **0.94** | **(0.92, 0.96)** | **0.93** | **(0.92, 0.95)** |
| ESR1_ CpG 9 | **0.92** | **(0.90, 0.94)** | **0.92** | **(0.90, 0.94)** |
| ESR1_ CpG 10 | **0.91** | **(0.89, 0.93)** | **0.90** | **(0.88, 0.93)** |
| ESR1_ CpG 11 | **0.96** | **(0.93, 0.99)** | **0.96** | **(0.93, 0.99)** |
| GFI1_region | **0.94** | **(0.92, 0.97)** | **0.94** | **(0.92, 0.97)** |
| GFI1_ CpG 1 | **0.95** | **(0.93, 0.96)** | **0.95** | **(0.93, 0.97)** |
| GFI1_ CpG 2 | **0.97** | **(0.95, 0.98)** | **0.97** | **(0.95, 0.98)** |
| GFI1_ CpG 3 | **0.96** | **(0.95, 0.98)** | **0.96** | **(0.95, 0.98)** |
| GFI1_ CpG 4 | 0.98 | (0.95, 1.00) | 0.98 | (0.95, 1.01) |
| GFI1_ CpG 5 | 0.98 | (0.94, 1.02) | 0.98 | (0.94, 1.03) |
| GFI1_ CpG 6 | 0.99 | (0.96, 1.01) | 0.99 | (0.96, 1.02) |
| GFI1_ CpG 7 | **0.96** | **(0.93, 0.99)** | **0.97** | **(0.94, 1.00)** |
| GFI1_ CpG 8 | 0.98 | (0.95, 1.00) | 0.98 | (0.96, 1.01) |
| GFI1_ CpG 9 | 0.99 | (0.95, 1.03) | 1.00 | (0.96, 1.04) |
| GFI1_ CpG 10 | 0.97 | (0.95, 1.00) | 0.98 | (0.95, 1.01) |
| GFI1_ CpG 11 | 0.98 | (0.95, 1.02) | 0.99 | (0.95, 1.03) |
| GFI1_ CpG 12 | **0.96** | **(0.94, 0.99)** | **0.97** | **(0.94, 1.00)** |
| GFI1_ CpG 13 | 0.98 | (0.95, 1.01) | 0.99 | (0.95, 1.02) |
| GFI1_ CpG 14 | **0.93** | **(0.92, 0.96)** | **0.94** | **(0.91, 0.96)** |
| GFI1_ CpG 15 | **0.94** | **(0.93, 0.96)** | **0.94** | **(0.93, 0.96)** |
| GFI1_ CpG 16 | **0.93** | **(0.91, 0.95)** | **0.93** | **(0.91, 0.95)** |
| GFI1_ CpG 17 | **0.93** | **(0.91, 0.95)** | **0.93** | **(0.91, 0.95)** |
| GFI1_ CpG 18 | **0.96** | **(0.94, 0.97)** | **0.96** | **(0.94, 0.98)** |
| GFI1_ CpG 19 | **0.94** | **(0.92, 0.97)** | **0.95** | **(0.92, 0.98)** |
| GFI1_ CpG 20 | **0.93** | **(0.91, 0.96)** | **0.93** | **(0.91, 0.96)** |
| GFI1_ CpG 21 | **0.96** | **(0.95, 0.98)** | **0.96** | **(0.95, 0.98)** |
| MYO1G_region | 1.01 | (0.99, 1.03) | 1.01 | (0.99, 1.03) |
| MYO1G_cluster1 | **0.97** | **(0.96, 0.99)** | **0.97** | **(0.96, 0.99)** |
| MYO1G_cluster2 | **1.04** | **(1.02, 1.06)** | **1.04** | **(1.02, 1.06)** |
| MYO1G_cluster3 | **0.95** | **(0.93, 0.98)** | **0.95** | **(0.93, 0.97)** |
| MYO1G_ CpG 1 | **0.97** | **(0.96, 0.98)** | **0.97** | **(0.95, 0.98)** |
| MYO1G_ CpG 2 | **0.98** | **(0.97, 0.99)** | **0.98** | **(0.97, 1.00)** |
| MYO1G_ CpG 3 | **1.03** | **(1.01, 1.05)** | **1.03** | **(1.01, 1.05)** |
| MYO1G_ CpG 4 | **1.02** | **(1.01, 1.04)** | **1.02** | **(1.01, 1.04)** |
| MYO1G_ CpG 5 | **1.02** | **(1.01, 1.04)** | **1.02** | **(1.01, 1.04)** |
| MYO1G_ CpG 6 | **1.02** | **(1.00, 1.04)** | **1.02** | **(1.00, 1.04)** |
| MYO1G_ CpG 7 | **1.03** | **(1.01, 1.04)** | **1.03** | **(1.01, 1.04)** |
| MYO1G_ CpG 8 | **1.03** | **(1.02, 1.05)** | **1.04** | **(1.02, 1.05)** |
| MYO1G_ CpG 9 | **1.03** | **(1.02, 1.05)** | **1.03** | **(1.02, 1.05)** |
| MYO1G_ CpG 10 | **1.03** | **(1.01, 1.05)** | **1.03** | **(1.01, 1.05)** |
| MYO1G_ CpG 11 | **1.02** | **(1.01, 1.04)** | **1.02** | **(1.01, 1.04)** |
| MYO1G_ CpG 12 | **1.04** | **(1.03, 1.06)** | **1.05** | **(1.03, 1.06)** |
| MYO1G_ CpG 13 | **1.06** | **(1.04, 1.07)** | **1.06** | **(1.04, 1.08)** |
| MYO1G_ CpG 14 | **0.98** | **(0.96, 0.99)** | **0.98** | **(0.96, 1.00)** |
| MYO1G_ CpG 15 | **0.94** | **(0.92, 0.96)** | **0.93** | **(0.91, 0.95)** |
| MYO1G_ CpG 16 | 0.98 | (0.96, 1.00) | **0.98** | **(0.95, 1.00)** |
| MYO1G_ CpG 17 | **0.97** | **(0.95, 0.99)** | **0.97** | **(0.95, 0.99)** |
| MYO1G_ CpG 18 | **0.95** | **(0.93, 0.97)** | **0.95** | **(0.92, 0.97)** |
| MYO1G_ CpG 19 | **0.95** | **(0.93, 0.97)** | **0.95** | **(0.93, 0.96)** |
| MYO1G_ CpG 20 | **0.95** | **(0.93, 0.97)** | **0.95** | **(0.93, 0.97)** |

Bold text indicates a statistically significant difference with a p-value < 0.05.

Adjusted for maternal cotinine levels, maternal age, family income, matrnal alcohol consumption during pregnancy, parity, child sex, pre-pregnancy BMI.

**Additional file 1: Table S3. Mediation analysis for the effect of DNA methylation in the association between active smoking during pregnancy and ADHD symptoms at 6 years of age.**

| **Mediator** | **Direct effect (c' path)** | | **Indirect effect** | |
| --- | --- | --- | --- | --- |
|  | **β** | **95 % CI** | **β** | **95 % CI** |
| AHRR_region | **0.66** | **(0.10, 1.22)** | -0.02 | (-0.28, 0.23) |
| AHRR_CpG1 | **0.72** | **(0.19, 1.24)** | -0.08 | (-0.23, 0.07) |
| AHRR_CpG2 | 0.35 | (-0.194, 0.89) | **0.29** | **(0.09, 0.50)** |
| AHRR_CpG3 | 0.52 | (-0.03, 1.08) | 0.11 | (-0.11, 0.34) |
| AHRR_CpG4 | **1.00** | **(0.43, 1.57)** | **-0.34** | **(-0.65, -0.09)** |
| AHRR_CpG5 | **0.73** | **(0.18, 1.29)** | -0.09 | (-0.35, 0.16) |
| CYP1A1_region | **0.63** | **(0.11, 1.14)** | 0.01 | (-0.07, 0.09) |
| CYP1A1_CpG1 | **0.59** | **(0.07, 1.10)** | 0.05 | (-0.04, 0.15) |
| CYP1A1_CpG2 | **0.59** | **(0.08, 1.10)** | 0.05 | (-0.05, 0.15) |
| CYP1A1_CpG3 | **0.68** | **(0.17, 1.20)** | -0.04 | (-0.14, 0.02) |
| CYP1A1_CpG4 | **0.61** | **(0.10, 1.12)** | 0.03 | (-0.03, 0.10) |
| CYP1A1_CpG5 | **0.62** | **(0.11, 1.14)** | 0.01 | (-0.06, 0.09) |
| CYP1A1_CpG6 | **0.63** | **(0.12, 1.14)** | 0.01 | (-0.03, 0.05) |
| CYP1A1_CpG7 | **0.63** | **(0.12, 1.14)** | 0.02 | (-0.01, 0.07) |
| CYP1A1_CpG8 | **0.67** | **(0.16, 1.18)** | -0.03 | (-0.10, 0.01) |
| CYP1A1_CpG9 | **0.63** | **(0.12, 1.14)** | 0.00 | (-0.04, 0.05) |
| CYP1A1_CpG10 | **0.64** | **(0.13, 1.15)** | 0.00 | (-0.04, 0.02) |
| CYP1A1_CpG11 | **0.64** | **(0.13, 1.15)** | 0.00 | (-0.04, 0.03) |
| CYP1A1_ CpG 12 | **0.63** | **(0.13, 1.14)** | 0.00 | (-0.02, 0.03) |
| CYP1A1_ CpG 13 | **0.64** | **(0.13, 1.15)** | 0.00 | (-0.02, 0.02) |
| CYP1A1_ CpG 14 | **0.66** | **(0.15, 1.17)** | -0.02 | (-0.09, 0.02) |
| CYP1A1_ CpG 15 | **0.61** | **(0.10, 1.12)** | 0.04 | (-0.01, 0.10) |
| CYP1A1_ CpG 16 | **0.60** | **(0.08, 1.11)** | 0.06 | (-0.02, 0.17) |
| CYP1A1_ CpG 17 | **0.68** | **(0.16, 1.19)** | -0.04 | (-0.22, 0.09) |
| ESR1_region | **0.61** | **(0.08, 1.13)** | 0.08 | (-0.05, 0.22) |
| ESR1_ CpG 1 | **0.65** | **(0.14, 1.15)** | -0.01 | (-0.05, 0.02) |
| ESR1_ CpG 2 | **0.63** | **(0.13, 1.14)** | 0.01 | (-0.02, 0.04) |
| ESR1_ CpG 3 | **0.59** | **(0.05, 1.12)** | 0.13 | (-0.04, 0.31) |
| ESR1_ CpG 4 | **0.63** | **(0.10, 1.15)** | 0.07 | (-0.07, 0.22) |
| ESR1_ CpG 5 | **0.60** | **(0.07, 1.13)** | 0.11 | (-0.05, 0.27) |
| ESR1_ CpG 6 | **0.58** | **(0.05, 1.10)** | 0.12 | (-0.03, 0.27) |
| ESR1_ CpG 7 | **0.58** | **(0.06, 1.11)** | 0.11 | (-0.04, 0.28) |
| ESR1_ CpG 8 | **0.59** | **(0.07, 1.12)** | 0.10 | (-0.04, 0.25) |
| ESR1_ CpG 9 | **0.64** | **(0.10, 1.17)** | 0.08 | (-0.10, 0.26) |
| ESR1_ CpG 10 | **0.64** | **(0.10, 1.18)** | 0.08 | (-0.11, 0.29) |
| ESR1_ CpG 11 | **0.63** | **(0.12, 1.15)** | 0.02 | (-0.04, 0.09) |
| GFI1_ CpG 1 | 0.31 | (-0.22, 0.84) | **0.31** | **(0.18, 0.48)** |
| GFI1_ CpG 2 | 0.31 | (-0.22, 0.85) | **0.33** | **(0.18, 0.51)** |
| GFI1_ CpG 3 | 0.30 | (-0.23, 0.83) | **0.34** | **(0.20, 0.53)** |
| GFI1_ CpG 4 | **0.55** | **(0.03, 1.08)** | 0.08 | (-0.06, 0.22) |
| GFI1_ CpG 5 | **0.59** | **(0.06, 1.11)** | 0.05 | (-0.08, 0.17) |
| GFI1_ CpG 6 | **0.60** | **(0.07, 1.13)** | 0.04 | (-0.11, 0.18) |
| GFI1_ CpG 7 | 0.49 | (-0.04, 1.02) | **0.14** | **(0.02, 0.28)** |
| GFI1_ CpG 8 | 0.54 | (0.00, 1.07) | 0.10 | (-0.06, 0.25) |
| GFI1_ CpG 9 | **0.63** | **(0.10, 1.15)** | 0.01 | (-0.15, 0.14) |
| GFI1_ CpG 10 | 0.53 | (0.00, 1.06) | 0.10 | (-0.05, 0.26) |
| GFI1_ CpG 11 | **0.60** | **(0.07, 1.13)** | 0.03 | (-0.13, 0.18) |
| GFI1_ CpG 12 | 0.49 | (-0.04, 1.02) | **0.14** | **(0.01, 0.28)** |
| GFI1_ CpG 13 | **0.57** | **(0.05, 1.10)** | 0.06 | (-0.07, 0.19) |
| GFI1_ CpG 14 | 0.29 | (-0.25, 0.83) | **0.33** | **(0.19, 0.52)** |
| GFI1_ CpG 15 | 0.23 | (-0.31, 0.77) | **0.39** | **(0.24, 0.59)** |
| GFI1_ CpG 16 | 0.27 | (-0.27, 0.81) | **0.35** | **(0.21, 0.54)** |
| GFI1_ CpG 17 | 0.31 | (-0.24, 0.84) | **0.31** | **(0.18, 0.49)** |
| GFI1_ CpG 18 | 0.38 | (-0.15, 0.91) | **0.25** | **(0.13, 0.40)** |
| GFI1_ CpG 19 | 0.43 | (-0.09, 0.96) | **0.19** | **(0.08, 0.34)** |
| GFI1_ CpG 20 | 0.35 | (-0.18, 0.88) | **0.27** | **(0.14, 0.43)** |
| GFI1_ CpG 21 | 0.37 | (-0.16, 0.90) | **0.27** | **(0.13, 0.43)** |
| MYO1G_region | **0.57** | **(0.05, 1.09)** | 0.04 | (-0.05, 0.14) |
| MYO1G_cluster1 | **0.77** | **(0.25, 1.23)** | **-0.16** | **(-0.27, -0.07)** |
| MYO1G_cluster2 | 0.46 | (-0.06, 0.98) | **0.18** | **(0.08, 0.31)** |
| MYO1G_cluster3 | **0.80** | **(0.27, 1.32)** | **-0.17** | **(-0.29, -0.09)** |
| MYO1G_ CpG 1 | **0.81** | **(0.28, 1.34)** | **-0.19** | **(-0.31, -0.10)** |
| MYO1G_ CpG 2 | 0.71 | **(0.19, 1.23)** | -0.10 | **(-0.20, -0.02)** |
| MYO1G_ CpG 3 | 0.51 | (-0.01, 1.03) | **0.11** | **(0.03, 0.22)** |
| MYO1G_ CpG 4 | 0.49 | (-0.03, 1.01) | **0.13** | **(0.04, 0.25)** |
| MYO1G_ CpG 5 | **0.52** | **(0.00, 1.04)** | **0.10** | **(0.02, 0.21)** |
| MYO1G_ CpG 6 | **0.52** | **(0.00, 1.04)** | **0.09** | **(0.01, 0.20)** |
| MYO1G_ CpG 7 | 0.51 | (-0.01, 1.03) | **0.12** | **(0.03, 0.23)** |
| MYO1G_ CpG 8 | 0.49 | (-0.03, 1.01) | **0.15** | **(0.06, 0.28)** |
| MYO1G_ CpG 9 | 0.49 | (-0.03, 1.01) | **0.14** | **(0.05, 0.25)** |
| MYO1G_ CpG 10 | **0.53** | **(0.01, 1.05)** | **0.10** | **(0.023, 0.20)** |
| MYO1G_ CpG 11 | 0.51 | (-0.01, 1.03) | **0.11** | **(0.03, 0.22)** |
| MYO1G_ CpG 12 | 0.49 | (-0.03, 1.01) | **0.17** | **(0.06, 0.31)** |
| MYO1G_ CpG 13 | 0.37 | (-0.16, 0.90) | **0.34** | **(0.19, 0.53)** |
| MYO1G_ CpG 14 | **0.69** | **(0.17, 1.21)** | **-0.09** | **(-0.18, -0.01)** |
| MYO1G_ CpG 15 | **0.86** | **(0.33, 1.39)** | **-0.21** | **(-0.36, -0.09)** |
| MYO1G_ CpG 16 | **0.68** | **(0.16, 1.20)** | -0.08 | (-0.17, 0.01) |
| MYO1G_ CpG 17 | **0.71** | **(0.18, 1.23)** | **-0.10** | **(-0.20, -0.02)** |
| MYO1G_ CpG 18 | **0.80** | **(0.27, 1.32)** | **-0.17** | **(-0.28, -0.08)** |
| MYO1G_ CpG 19 | **0.84** | **(0.31, 1.37)** | **-0.20** | **(-0.31, -0.11)** |
| MYO1G_ CpG 20 | **0.78** | **(0.25, 1.30)** | **-0.15** | **(-0.25, -0.07)** |

Bold text indicates a statistically significant difference with a p-value < 0.05.

Adjusted for maternal age, family income, matrnal alcohol consumption during pregnancy, parity, child sex, pre-pregnancy BMI.

**Additional file 1: Table S4. List of bisulphite PCR primers.**

| **Name** | **Primer sequence** |
| --- | --- |
| AHRR_Fwd | TATTTTTGAGAGGGTAGTTTTGTTT |
| AHRR_Rev | AACCACTCCCAAAACCCAC |
| CYP1A1_Fwd | GTTATGTTAAATGGTATTGGGGTTT |
| CYP1A1_Rev | AAACTCTTAAAAAACCAACCTC |
| ESR1_Fwd | TGTTAGTTATGATGATGATTTTTTGG |
| ESR1_Rev | AATTATTTTTAAACACTTATACTTCCATAT |
| GFI1_Fwd | TTGTTTATTGTTTTGTTTTTTATAG |
| GFI1_Rev | ATAAACACATTAACTTCTCC |
| MYO1G_Fwd | TTTAGAATTATTAGTTTTGGAGAAAAT |
| MYO1G_Rev | TACAACACCACCAACTAATCTCCT |
